# Eurotatorian paraphyly: Revisiting phylogenetic relationships based on the complete mitochondrial genome sequence of *Rotaria rotatoria *(Bdelloidea: Rotifera: Syndermata)

**DOI:** 10.1186/1471-2164-10-533

**Published:** 2009-11-17

**Authors:** Gi-Sik Min, Joong-Ki Park

**Affiliations:** 1 Department of Biological Sciences, Inha University, Incheon 402-751, Republic of Korea; 2 Department of Parasitology, College of Medicine, Chungbuk National University, Cheongju 361-763, Republic of Korea

## Abstract

**Background:**

The Syndermata (Rotifera+Acanthocephala) is one of the best model systems for studying the evolutionary origins and persistence of different life styles because it contains a series of lineage-specific life histories: Monogononta (cyclic parthenogenetic and free-living), Bdelloidea (entirely parthenogenetic and mostly benthic dweller), Seisonidea (exclusively bisexual and epizoic or ectoparasitic), and Acanthocephala (sexual and obligatory endoparasitic). Providing phylogenetic resolution to the question of Eurotatoria (Monogononta and Bdelloidea) monophyly versus paraphyly is a key factor for better understanding the evolution of different life styles, yet this matter is not clearly resolved. In this study, we revisited this issue based on comparative analysis of complete mitochondrial genome information for major groups of the Syndermata.

**Results:**

We determined the first complete mitochondrial genome sequences (15,319 bp) of a bdelloid rotifer, *Rotaria rotatoria*. In order to examine the validity of Eurotatoria (Monogononta and Bdelloidea) monophyly/paraphyly, we performed phylogenetic analysis of amino acid sequences for eleven protein-coding genes sampled from a wide variety of bilaterian representatives. The resulting mitochondrial genome trees, inferred using different algorithms, consistently failed to recover Monogononta and Bdelloidea as monophyletic, but instead identified them as a paraphyletic assemblage. Bdelloidea (as represented by *R. rotatoria*) shares most common ancestry with Acanthocephala (as represented by *L. thecatus*) rather than with monogonont *B. plicatilis*, the other representative of Eurotatoria.

**Conclusion:**

Comparisons of inferred amino acid sequence and gene arrangement patterns with those of other metazoan mtDNAs (including those of acanthocephalan *L. thecatus *and monogonont *B. plicatilis*) support the hypothesis that Bdelloidea shares most common ancestry with Acanthocephala rather than with Monogononta. From this finding, we suggest that the obligatory asexuality of bdelloideans may have secondarily derived from some other preexisting condition in earlier lineage of rotifers. Providing a more complete assessment of phylogenetic relationships and inferring patterns of evolution of different types of life styles among Syndermata awaits comparisons requiring mitochondrial genome sequencing of Seisonidea.

## Background

The Rotifera (also called Rotatoria) is a group of aquatic micrometazoans, mostly less than a millimeter in size. It includes more than 2,000 described species that usually occur in freshwater and marine environments throughout the world, but some species are found in wet terrestrial habitats, such as moist soil, or mosses and lichens living on fallen trees and rocks [1-3]. This group is usually distinguished from other metazoans by presence of the corona ("wheel organ" = the crown of cilia) located in the cephalic region, which is used for locomotion and food gathering. Due to their great abundance and high reproductive potential, rotifers have been considered to play a significant role in the food webs of certain freshwater environments through their involvement in energy cycling and nutrient transfer [4-6]. Most authorities accept that the Rotifera consists of three classes, each having a unique reproductive strategy [2,7,8]: Bdelloidea (exclusively parthenogenetic), Monogononta (cyclic parthenogenetic with facultative sexual reproduction), and Seisonidea (exclusively bisexual). Of these, the class Bdelloidea is very unique in that its species (several hundred) are exclusively female. This is the largest metazoan asexual group where no sexual reproduction has ever been reported and represents an ancient origin of asexuality with great evolutionary success in the diversification of the species [9,10]. The class Monogononta, representing the largest group of rotifers, comprises more than 1,500 species. They are mostly found in freshwater, brackish and marine waters and are characterized by a unique life cycle, cyclical parthenogenesis, that alternates between two reproductive modes, i.e. amictic and mictic phases of reproduction according to the absence/presence of males, respectively [2,5]. The class Seisonidea consists of three epizoic species (belong to two genera, *Seison *and *Paraseison *[11]) on marine crustaceans (*Nebalia *species) and all of these are known to reproduce by amphimixis [12].

There is little doubt about the close relationship between Rotifera and Acanthocephala, the clade known as Syndermata because they share the feature of the syncytial epidermis [13,14]. This affinity has received broad support from many earlier studies utilizing different sources of phylogenetic information: morphology [15-17], SSU rDNA sequences [18-20], combined analysis of molecular and morphological characters [21,22], and combined analysis of SSU and LSU ribosomal DNA sequences [23]. In contrast to syndermatan monophyly, internal phylogeny within the clade (among Bdelloidea, Monogononta, Seisonidea, and Acanthocephala) has been the subject of relatively vigorous contention [24-27]. Most controversies are related to Eurotatorian monophyly versus paraphyly, although there are differences regarding the phylogenetic position of Seisonidea within the clade. The monophyly/non-monophyly of eurotatorians (Monogononta and Bdelloidea) relative to Acanthocephala has received much attention because this issue plays a key role in understanding the evolution and ecological diversity found among major groups of Syndermata. Lorenzen [15] first recognized the lemnisci (paired projections of the neck epidermis into the body cavity) and proboscis (an invaginable anterior part of the body) as synapomorphic characters for unifying the Bdelloidea and Acanthocephala (inferred the latter as being the highly specialized sister group to the former; see [24] for different view), and this relationship has also been recovered in many subsequent molecular analyses (SSU+LSU+mtDNA cox1 [26]; SSU [19]; SSU+mtDNA 16S [20]; SSU+LSU+histone H3+mtDNA cox1[28]). Based on morphological and molecular perspectives, Garey et al. [19] recognized Acanthocephala as a subtaxon of Rotifera and united these two groups under the superclass 'Lemniscea' (Lemniscea hypothesis-eurotatorian paraphyly). However, eurotatorian monophyly (Monogononta+Bdelloidea) and sister-group relationship of Seisonidea and Acanthocephala have also been suggested from some earlier studies based on morphological evidence (ultrastructural similarity [14,29]; cladistic analysis of morphological dataset [7,30,31]) or from molecular analysis (the partial sequence of nuclear heat-shock protein *hsp82 *[8]; SSU rDNA [32]; combined analysis of *hsp82*+SSU data [33]). Furthermore, employing different methods for phylogenetic analysis resulted in inconsistent tree topologies when SSU data were analyzed [34]. Although the most recently published work from EST-based phylogenomic analysis supported Eurotatoria paraphyly [27], but the phylogenetic issue regarding the Eurotatoria monophyly/paraphyly still awaits other types of data for corroboration.

With a very few exceptions, metazoan mitochondrial genomes are circular DNA molecules (mostly less than 16 kb in size) that encode 37 genes: 13 protein-coding genes (*atp8 *is missing in many nematode and flatworm species so far reported), two ribosomal RNA genes, and 22 transfer RNA genes [35,36]. Due to its universality and remarkably stable feature in genome content across various metazoan phyla, comparisons of the mitochondrial genome information (e.g., nucleotide sequence, amino acid sequence and gene order rearrangement) have often proven useful for reconstructing the deep node phylogeny and for assessing the phylogenetic relationships among closely related species [36-39]. In recent years, there has been an unprecedented increase in mitochondrial genomic surveys in relation to phylogenetic comparisons of a variety of animal groups. Mitochondrial genome information has now become available for more than a thousand animal species (See NCBI metazoan mitochondrial genome resources). However, the distribution of completely characterized mitochondrial genome sequences has been strongly biased across the metazoan taxa: the subphylum Vertebrata and the phylum Arthropoda account for more than 80% of the metazoan mitochondrial genome data determined so far, whereas there are still a considerable number of metazoan phyla for which there is limited mitochondrial genome information, and some phyla have never been investigated [40,41]. Complete mitochondrial genome sequencing from poorly investigated groups is needed to supplement a gap in our current understanding of mitochondrial DNA evolution in the metazoa.

Mitochondrial genome information from the Syndermata was reported, for the first time, from the acanthocephalan species *Leptorhynchoides thecatus *[42] and recently thereafter from the monogonont rotifer *Brachionus plicatilis *[43]. Despite belonging to the same clade (Syndermata), the mitochondrial genomes reported for these two organisms did not share many characteristics in their organization such as chromosome structure, gene order, codon usage and the secondary structure of tRNA molecules. For example, the mitochondrial genome of *B. plicatilis*, a representative of the Monogononta, is encoded in two separate mitochondrial chromosomes, each having different gene content and copy number. In contrast, as in most metazonas, all genes of *L. thecatus *mtDNA are encoded in a single type of circular mitochondrial DNA molecule. The lack of common features in the mitochondrial genomes between these species necessitates additional characterization of mitochondrial genomes from the other major groups of the syndermata. Information from Bdelloidea is expected to supplement our understanding of syndermatan mitochondrial genome evolution and provide utility as a molecular marker in resolving internal phylogeny among the major groups of the Syndermata. To this end, we characterized the first complete mitochondrial genome sequence of the bdelloid species *Rotaria rotatoria*, and compared its mitochondrial genome information with other syndermatan species in order to investigate phylogenetic issues regarding the monophyly or paraphyly of Eurotatoria.

## Results and Discussion

### General features of the *R. rotatoria *mitochondrial genome

**Figure 1:**
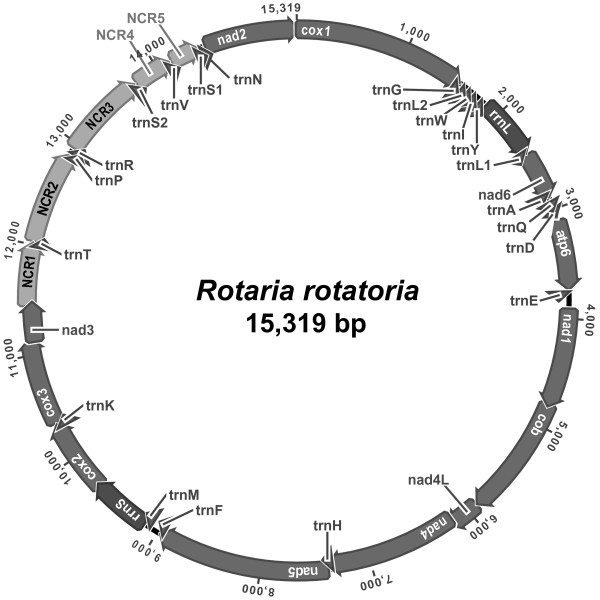
**Circular representation of the mitochondrial genome of *Rotaria rotatoria***. All genes are encoded in the same direction and 21 tRNA genes are denoted by the one-letter abbreviation and two leucine and two serine tRNA genes are labeled, according to their anticodon sequence, as L1 (*trnL-uag*), L2 (*trnL-uaa*), S1 (*trnS-ucu*), and S2 (*trnS-uga*), respectively. The intergenic non-coding regions with notable size (>200 bp) are denoted as NCR1-NCR5, respectively.

The mitochondrial genome of *R. rotatoria *is 15,319 bp in length (GenBank accession no. GQ304898) and contains 12 protein-coding genes (lacking *atp8*), two rRNA genes, and as is occasionally found in some other metazoans, all but one (*trnC*) of the typical 22 tRNAs genes. All genes are encoded in the same direction in a single circular mitochondrial DNA (Fig. 1), unlike that of the monogonont rotifer *B. plicatilis*, in which mitochondrial genes are encoded in two separated circular chromosomes [43]. The genome organization including the gene order, length of overlapping regions between the genes and length of intergenic spacer regions is shown in Table 1. The nucleotide composition of the entire *R. rotatoria *mtDNA sequence is 29.5% A, 43.7% T, 17.4% G, and 9.5% C (Table 2). The overall A+T content (73.1%) approximates more or less that of the acanthocephalan *L. thecatus *(71.5% in A+T content), but is significantly higher than those found in the monogonont rotifer *B. plicatilis *(A+T contents of 63.9% and 62.9% for mtDNA-I and mtDNA-II, respectively). The average of A+T composition of protein-coding genes (73.7%) is very similar to that of the entire sequence, but there is noticeable compositional difference across the codon positions: the average A+T content of the third position (80.9%) is noticeably higher than those of the first (67.8%) and second codon (72.4%) positions, indicating that there is a strong bias toward T (47.2%) and A (33.7%) in third codon position.

**Table 1:** The mitochondrial genome organization of *Rotaria rotatoria*

Gene/ region	Positions		Size		Codons		Intergenic nucleotides
				
	Start	End	No. of nt	No. of aa	Initiation	Termination	
*cox1*	1	1542	1542	513	ATT	TAA	-5
*trnG*	1538	1594	57				24
*trnL2*	1619	1675	57				5
*trnW*	1681	1734	54				6
*trnI*	1741	1805	65				24
*trnY*	1830	1893	64				0
*rrnL*	1894	2422	529				0
*trnL1*	2423	2478	56				-6
*nad6*	2473	2862	390	129	ATT	TAG	1
*trnA*	2864	2921	58				18
*trnQ*	2940	2994	55				-1
*trnD*	2994	3044	51				76
*atp6*	3121	3717	597	198	ATG	TAG	48
*trnE*	3766	3824	59				100
*nad1*	3925	4797	873	290	ATT	TAA	0
*cob*	4798	5883	1086	361	ATG	TAA	42
*nad4L*	5926	6198	273	90	ATG	TAA	-7
*nad4*	6192	7364	1173	390	ATA	TAA	-1
*trnH*	7364	7417	54				-12
*nad5*	7406	8947	1542	513	ATT	TAA	2
*trnF*	8950	9006	57				80
*trnM*	9087	9147	61				0
*rrnS*	9148	9668	521				0
*cox2*	9669	10317	649	216	ATG	T	0
*trnK*	10318	10369	52				-3
*cox3*	10367	11134	768	255	ATA	TAA	-1
*nad3*	11134	11466	333	110	ATG	TAA	535 (NCR1)
*trnT*	12002	12056	55				806 (NCR2)
*trnP*	12863	12915	53				7
*trnR*	12923	12969	47				760 (NCR3)
*trnS2*	13730	13786	57				316 (NCR4)
*trnV*	14103	14155	53				236 (NCR5)
*trnS1*	14392	14440	49				0
*trnN*	14441	14493	53				-6
*nad2*	14488	15313	826	275	ATG	T	6

**Table 2:** Nucleotide composition of the mitochondrial genome of *Rotaria rotatoria*

Nucleotide		Length (bp)	T (%)	C (%)	A (%)	G (%)	A+T (%)	G+C (%)
Entire sequence		15319	43.7	9.5	29.5	17.4	73.1	26.9
Protein-coding sequence^§^		10020	46.1	8.6	27.5	17.7	73.7	26.3
Codon position	1st	3340	37.4	8.8	30.4	23.3	67.8	32.1
	2nd	3340	53.8	11.5	18.5	16.1	72.4	27.6
	3rd	3340	47.2	5.4	33.7	13.7	80.9	19.1
Ribosomal RNA gene sequence		1050	39.6	7.6	37.0	15.8	76.6	23.4
Transfer RNA gene sequence		1167	43.0	7.4	33.7	15.9	76.7	23.3
Non-coding regions (NCRs)		2653	36.6	15.1	30.9	17.3	67.5	32.5
	NCR1	535	39.1	14.2	31.4	15.3	70.5	29.5
	NCR2	806	35.4	14.4	31.8	18.5	67.1	32.9
	NCR3	760	29.3	20.8	33.3	16.6	62.6	37.4
	NCR4	316	46.8	9.5	24.1	19.6	70.9	29.1
	NCR5	236	45.3	8.9	28.4	17.4	73.7	26.3

^§ ^Termination codons were excluded

### Protein-coding genes

Twelve protein-coding genes were identified using NCBI ORF Finder and by comparing their sequence with those of homologous genes reported from the acanthocephalan *L. thecatus *[42] and monogonont *B. plicatilis *[43]. Even after an exhaustive search using BLAST, we failed to discover any *atp8*-like protein sequence. The lack of *atp8 *in the mtDNA genome is not very rare, and quite common in most nematode and platyhelminth species reported so far (cf. *Trichinella spiralis*, a nematode species where the *atp8 *exists [44]).

In many metazoan mtDNAs, the codon usage is strongly biased, especially in the third position of synonymous codons within amino acid families [35,45-47]. In the protein-coding genes of *R. rotatoria *mtDNA, the higher A+T content is related to amino acid composition with higher abundance of A+T-rich codons. Amino acids encoded by T-rich and A-rich codons (more than two Ts and As in a triplet, respectively) account for 47.3% and 22.2% of the total amino acid composition, respectively, totaling 69.5% of the entire protein sequences (Table 3). Another factor that causes nucleotide bias is differential preference of thymine (T) or adenine (A) in the third position of codons. The average relative nucleotide frequency in the third position is 47.2% (T), 33.7% (A), 13.7% (G), and 5.4% (C), respectively. Indeed, both in twofold- and fourfold-degenerate codon families, the T-ending codons are greatly favored in most synonymous codons, whereas C-ending codons are evidently avoided. The relative frequency of TTT (9.5%) coding phenylalanine is much higher than that of its degenerate codon TTC (1.5%). Another notable example of biased codon usage is that the codon ATT (8.0%) is much more frequently used than ATC (0.7%) in the isoleucine family. The propensity of a preference for T, with an apparent bias against C may reflect mutational bias at the third codon position as postulated in other metazoans [48]. Of 12 protein-coding genes, six (*atp6, cob, nad4L, cox2, nad3*, and *nad2*) use ATG as the initiation codon, whereas others initiate with ATT (*cox1, nad6, nad1*, and *nad5*), or ATA (*nad4 *and *cox3*), respectively (Table 1). The majority of genes end with complete termination codon TAA (*cox1, nad1, cob, nad4L, nad4, nad5, cox3*, and *nad3*) or TAG (*nad6 *and *atp6*), but *cox2 *and *nad2 *terminate with the incomplete stop codon T, which is presumed to become a complete termination codon through post-transcriptional polyadenylation [49].

**Table 3:** Codon usage for 12 protein-coding genes of the mitochondrial genome of *Rotaria rotatoria*

Codon (aa)	nc	%	Codon (aa)	nc	%	Codon (aa)	nc	%	Codon (aa)	nc	%
TTT(F)	319	9.5	TCT(S)	105	3.1	TAT(Y)	157	4.7	TGT(C)	32	1.0
TTC(F)	50	1.5	TCC(S)	7	0.2	TAC(Y)	20	0.6	TGC(C)	2	0.1
TTA(L)	367	11.0	TCA(S)	20	0.6	TAA(*)	8	0.2	TGA(W)	62	1.9
TTG(L)	79	2.4	TCG(S)	6	0.2	TAG(*)	2	0.1	TGG(W)	24	0.7
CTT(L)	58	1.7	CCT(P)	49	1.5	CAT(H)	49	1.5	CGT(R)	27	0.8
CTC(L)	3	0.1	CCC(P)	5	0.1	CAC(H)	1	0.0	CGC(R)	1	0.0
CTA(L)	42	1.3	CCA(P)	8	0.2	CAA(Q)	22	0.7	CGA(R)	7	0.2
CTG(L)	6	0.2	CCG(P)	4	0.1	CAG(Q)	12	0.4	CGG(R)	1	0.0
ATT(I)	267	8.0	ACT(T)	55	1.6	AAT(N)	92	2.7	AGT(S)	36	1.1
ATC(I)	23	0.7	ACC(T)	0	0.0	AAC(N)	13	0.4	AGC(S)	8	0.2
ATA(M)	180	5.4	ACA(T)	22	0.7	AAA(K)	56	1.7	AGA(S)	90	2.7
ATG(M)	74	2.2	ACG(T)	6	0.2	AAG(K)	39	1.2	AGG(S)	55	1.6
GTT(V)	147	4.4	GCT(A)	61	1.8	GAT(D)	68	2.0	GGT(G)	54	1.6
GTC(V)	19	0.6	GCC(A)	6	0.2	GAC(D)	9	0.3	GGC(G)	15	0.4
GTA(V)	126	3.8	GCA(A)	20	0.6	GAA(E)	44	1.3	GGA(G)	59	1.8
GTG(V)	38	1.1	GCG(A)	11	0.3	GAG(E)	36	1.1	GGG(G)	66	2.0

The letter in parenthesis indicates one-letter abbreviation of the amino acids.

* Stop (termination) codon.

### Transfer RNA and ribosomal RNA genes

Implementation of tRNAscan algorithm failed to find any tRNA-like secondary structure, but we manually identified them by eye. Of 22 tRNA genes normally found in most other metazoan mtDNAs, 21 tRNA-like nucleotide segments (ranging from 47 to 65 bp in size) except for *trnC *can be folded into a cloverleaf secondary structure with some mismatches or incomplete configuration of the DHU and/or TΨC arms (Additional file 1). In spite of the exhaustive tRNA search, we were not able to identify any candidates for *trnC *with high confidence. Out of 21 tRNAs found, only 10 (*trnA, trnL1, trnM, trnF, trnP, trnS1, trnS2, trnT, trnY*, and *trnV*) display a typical cloverleaf-like structure equipped with DHU and TΨC arms. Many other tRNAs appear to lack a TΨC arm (6 tRNAs; *trnD, trnQ, trnE, trnG, trnH*, and *trnK*) or a DHU arm (4 tRNAs; *trnN, trnI, trnL2*, and *trnW*), or both (1 tRNAs; *trnR*). The rarity of having a typical cloverleaf-like secondary structure was also found in the acanthocephalan species *Leptorhynchoides thecatus *[42] where almost none of the inferred tRNAs displayed a typical stem-and-loop configuration in the DHU and TΨC arms. This differs from most other metazoan mtDNAs, including the monogonont rotifer *Brachionus plicatilis *[43]. Indeed, the putative secondary structure of 22 tRNAs found in *B. plicatilis *contains a typical cloverleaf structure comprising a amino-acyl arm (a stem of seven nucleotide pairs; ntp), a DHU arm (a stem of 2-4 ntp with a 2-8 nt loop), an anticodon arm (a stem of 5 ntp with an anticodon) and a TΨC arm (a stem of 2-4 ntp with a 2-7 nt loop).

Based on sequence comparison with *L. thecatus *and *B. plicatilis*, two ribosomal RNA genes were identified: The *rrnL *(529 bp) is located between *trnY *and *trnL1 *as found in *L. thecatus*, but its size is remarkably smaller than any other metazoan large ribosomal subunit molecule (mostly larger than 1 kb) including those of other syndermatans reported thus far (e.g., 925 bp and 1,107 bp in *L. thecatus *and *B. plicatilis*, respectively). The *rrnS *(521 bp) is located between *trnM *and *cox2*.

### Non-coding regions

A total of 19 intergenic non-coding sequences, ranging from 1 to 806 bp in size (3,092 bp in total accounting for 20.2% of entire genome length), were detected. Of these, five intergenic non-coding regions (NCR) are prominent by their significant lengths (≥ 200 bp), ranging from 236 bp to 806 bp (535 bp-NCR1 [between *nad3 *and *trnT*], 806 bp-NCR2 [between *trnT *and *trnP*], 760 bp-NCR3 [between *trnR *and *trnS2*], 316 bp-NCR4 [between *trnS2 *and *trnV*], and 236 bp-NCR5 [between *trnV *and *trnS1*, respectively). Despite an exhaustive search using the NCBI ORF Finder, we were not able to find ORF-like candidates of significant length from these spacer regions. The relatively larger genome size of the *R. rotatoria *mtDNA is attributed to the abundance of conspicuously long, unassigned spacer regions, compared to that of acanthocephalan *L. thecatus *(13,888 bp [42]). The region located between *trnR *and *trnS2 *(NCR3) contains 24 tandemly repeated units of a 18-nt sequences (5'-NRRWYBTYRNGHRRYYYY-3'), each having potential to be folded into a stem-loop structure (a stem of 5-6 ntp with a 6-8 nt loop). Such repeats, known as variable numbers of tandem repeats (VNTRs), have been reported from a variety of animal mtDNAs and they have been utilized as molecular markers for detecting subdivision of the population in ecological studies (See [50] for a review).

### Mitochondrial molecular phylogeny

#### Phylogenetic position of Platyzoa within Lophotrochozoa

**Figure 2:**
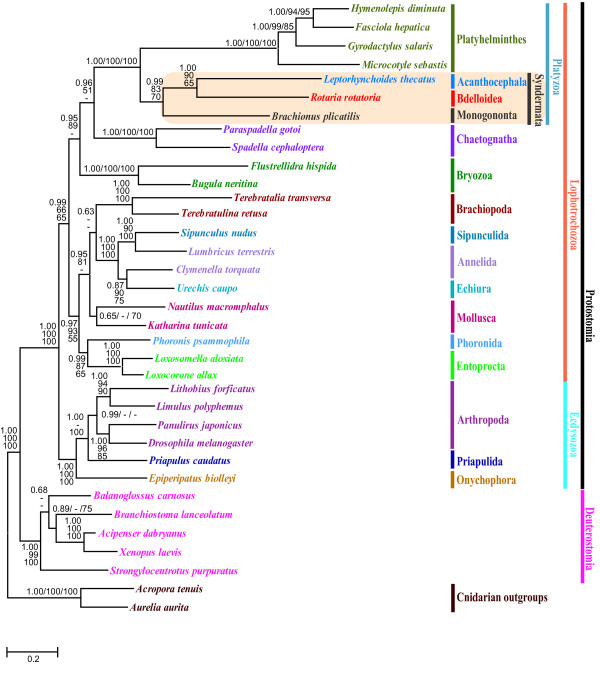
**Mitochondrial gene tree from Bayesian analysis showing the phylogenetic relationships among 35 metazoan species**. The tree topologies from Bayesian analysis and maximum likelihood are very similar. Numbers above/below branches are Bayesian posterior probability (BPP) and local rearrangement-expected likelihood weight (LR-ELW) edge support, and bootstrap (BP) values from maximum likelihood analysis, respectively (BPP/LR-ELW/BP). The branches that are supported with values of ≤50% or not consistent in their positions between Bayesian and maximum likelihood methods are represented by "-".

The Platyzoa refers to an assemblage of some acoelomate and pseudocoelomate animal taxa including Platyhelminthes, Rotifera, Acanthocephala, Gastrotricha, and Gnathostomulida [51]. Phylogenetic affinity of these groups with other lophotrochozoans has not yet been confirmed. Results of recent molecular analyses depicted Platyzoa as a sister to Trochozoa (morphology+SSU dataset [21]), or as a derived subclade of Lophotrochozoa (LSU+SSU dataset [23]. This disagreement still awaits unambiguous resolution. In this study, we performed phylogenetic analyses for the amino acid dataset of major representatives of Bilateria with two cnidarian sequences (*Aurelia aurita *and *Acropora tenuis*) as outgroups. Resulting trees from both ML and BI methods showed that the protostomes and deuterostomes were divided into two separate clades with very high support (Fig. 2). Within the protostome clade, monophyly of Ecdysozoa and monophyly of Lophotrochozoa were also found, but the dichotomy between these two groups was collapsed when the nematode sequences (*Caenorhabditis elegans, Trichinella spiralis*) were included in the analysis. In all cases, nematode sequences were nested within the Lophotrochozoan clade rather than grouped with ecdysozoan members (Additional file 2), and their position varied considerably according to different phylogenetic methods. Their unusual position appears to be artificial and due to the long-branch attraction (LBA) problem as documented in many previous papers [52-54]. Therefore, in order to reduce this factor in the tree reconstruction, we excluded nematode sequences from subsequent analyses. After the exclusion of nematodes, the concatenated amino acid sequence dataset consisting of 1,556 homologous positions was used for the subsequent analyses. Note that monophyly of Lophotrochozoa and monophyly of Ecdysozoa were recovered after removal of nematode sequences from the analyses, and support values for clades were noticeably improved in all analyses (see table description of Additional file 3 for the phylogenetic position of Chaetognatha within the metozoa). The monophyletic grouping of ecdysozoan members received 100% BP (bootstrap percentage) in ML, and 1.00 BPP (Bayesian posterior probability). The Lophotrochozoan clade was also well supported with 1.00 BPP in BI, but received a very weak bootstrap support value of 56% in ML analysis (Fig. 2). Although the Lophotrochozoan clade is not strongly supported, the current amino acid sequence data support the Ecdysozoa/Lophotrochozoa split. It is particularly noteworthy that both phylogenetic methods (BI and ML) recovered Platyhelminthes, Rotifera, and Acanthocephala (all platyzoan members included in this study) as monophyletic lineages in the Lophotrochozoan clade and nodal support for Platyzoa was very strong (100% in both BI and ML analysis). This result is in agreement with an earlier analysis based on a combined dataset of LSU and SSU sequences that recognized the Platyzoa as a derived monophyletic assemblage within the Lophotrochozoa [23]. As yet, the availability of complete mitochondrial genome information is limited to Platyhelminthes, Acanthocephala, and Rotifera, and further evidence from Gnathostomulida and Gastrotricha is required to confirm the monophyly of Platyzoa and its derived position within the Lophotrochozon clade (sensu [51]).

#### Phylogenetic implications for Syndermata: Eurotatoria paraphyly

Phylogenetic relationships among Syndermata, in particular with regard to Eurotatoria monophyly/paraphyly are still vigorously debated. The answer to this question is prerequisite for correct understanding of the evolution of different types of life styles found among major groups of Syndermata [27]. In this study, we revisited this issue based on phylogenetic analysis of amino acid sequence for eleven protein-coding genes (except *atp6 *and *atp8*) of the complete mitochondrial genome. The resulting trees both from Bayesian and maximum likelihood analyses recovered syndermatan members (Monogononta, Bdelloidea, and Acanthocephala) as a monophyletic group, which is in turn grouped with Platyhelminthes (Fig. 2). The phylogenetic affinity of the Syndermata with Platyhelminthes in this study is concordant with some earlier publications asserting their sister-relationship [23,55]. It is particularly significant to note that both BI and ML methods placed Bdelloidea and Acanthocephala as sister taxa and this relationship was very strongly supported by 1.00 BPP in BI analysis, but received relatively weaker support (65% BP) in ML analysis. Moreover, none of the phylogenetic methods recovered the two eurotatorian species, *Rotaria rotatoria *(Bdelloidea) and *Brachionus plicatilis *(Monogononta) as monophyletic, but instead always depicted them as a paraphyletic assemblage. Bdelloidea represented by *R. rotatoria *appears to share most recent common ancestry with Acanthocephala rather than with the monogonont *B. plicatilis*. The sister-group position of the Monogononta to the Bdelloidea+Acanthocephala, received strong support from BI (0.99 BPP) and moderate support from ML (70% BP), respectively. This result indicates a firm support for the 'Eurotatorian paraphyly' hypothesis. In all cases of the analysis, monophyly of Bdelloidea + Acanthocephala relative to Monogononta is always very robust. In order to assess whether our data rejects the alternative hypothesis (Eurotatoria monophyly), we performed statistical comparison of the likelihood scores for the best ML tree without constraint versus the best tree with the topological constraint of Eurotatoria monophyly using theTreefinder program. The result of topology test failed to detect a significant difference between these two competing hypotheses under various criteria (ELW, SH and AU; Table 4). Although Eurotatoria monophyly was not significantly worse interpretation of these data by tree topology comparison test, the optimal phylogenetic trees from different methods unanimously depicted *B. plicatilis *(Monogononta) sister to two other syndermatans (bdelloidean *R. rotatoria *and acanthocephalan *L. thecatus*), regardless of different options applied in the data analysis (including or excluding inferred gaps in the analysis). In addition, this relation (Eurotatoria paraphyly) is further corroborated by sharing greater similarity in gene arrangement between *R. rotatoria *and *L. thecatus *(next section).

**Table 4:** Results of tree topology test using different criteria: expected likelihood weights (ELW), Shimodaira-Hasegawa (SH), and approximately unbiased (AU)

Hypothesis	*-ln*	*Δ -ln*	ELW	SH	AU
Best ML tree	56700.07				
Monophyly of Eurotatoria	56724.49	24.42	0.199	0.197	0.196

**Figure 3:**
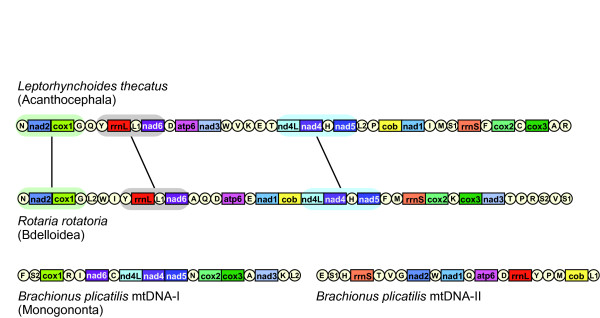
**Linearized comparison of the mitochondrial gene arrangement of three syndermatan species**. Gene and genome size are not to scale. All genes are transcribed in the same direction (from left to right). The tRNAs are labeled by single-letter abbreviations. Gene clusters shared between *L. thecatus *(Acanthocephala) and *R. rotatoria *(Bdelloidea) are represented by shadowed areas.

A close relationship between Bdelloidea and Acanthocephala is also evident when gene arrangement patterns of *R. rotatoria *mtDNA are compared with other metazoan animal groups. Gene arrangement of *R. rotatoria *mtDNA is very different from those found in other metazoans, but shows the highest similarity to the acanthocephalan *L. thecatus*. The gene order comparison between *R. rotatoria *and *L. thecatus *identified the gene order *of trnN-nad2-cox1-trnG, trnY-rrnL-trnL1-nad6*, and *nad4L-nad4-trnH-nad5 *shared between the representatives of the Bdelloidea and Acanthocephala, respectively (Fig. 3). The gene order of *nad4L-nad4-trnH-nad5 *can also be found across many various metazoan mtDNAs, such as Bryozoa [56], Brachiura (Arthropoda [57]), Gastropoda (Mollusca [58]), Vertebrata (Teleostei [59] and Amphibia [60]), and Priapulida [54]. However, this conserved gene order is not shared by the *B. plicatilis *mtDNA and we suggest it is due to the idiosyncratic features of *B. plicatilis *mtDNA in which 36 genes are encoded separately into bipartite mitochondrial genomes [43]. In contrast, note that the gene order similarity found between *R. rotatoria *and *L. thecatus *(*trnN-nad2-cox1-trnG *and *trnY-rrnL-trnL1-nad6*) is very unique among the metazoans and this shared gene rearrangement lends another line of strong support for their common ancestry. The phylogenetic conclusion of Eurotatoria paraphyly suggested from the present study is also consistent with the most recent EST based phylogenomic analysis [27].

In general, the evolutionary potential for adaptive radiation and its subsequent diversification of extant biota are often associated with preferential adoption of different life styles during evolutionary history. In most cases, a robust phylogeny is a prerequisite to correctly understand the evolution of life styles among the groups. The Syndermata (Rotifera+Acanthocephala) is one of the best model systems for studying the evolutionary origin and persistence of different life styles because it shows a variety of lineage-specific reproduction modes. Of these, Bdelloidea is particularly among the most unique in that it contains exclusively several hundred species and all individuals are females, the largest metazoan group with no sexual reproduction that has ever been documented [9]. This group is also considered to represent an ancient origin of asexuality with a great evolutionary success in their diversification of asexual species [9,10]. There are some additional cytological [61] and molecular evidence [62] suggesting that the evolution and maintenance of asexuality in Bdelloidea is of relatively ancient origin, dating back to more than tens of millions of years ago. Inferring the history of the obligatory asexual class Bdelloidea within the syndermatan phylogenetic framework is a central topic in evolutionary biology of asexuality because these organisms have long been regarded as perfect candidates to test the advantage of sexual reproduction [63]. The phylogenetic conclusion obtained from this comparative mitochondrial genome study provides essential clues in extrapolating the directionality of long-term asexual evolution of bdelloidean lineage. The sister group position of Monogononta to the Bdelloidea/Acanthocephala clade suggests that the obligatory asexuality of bdelloideans may have been derived secondarily from some other preexisting condition in earlier lineage of rotifers. However, because no mitochondrial genome information is available from Seisonidea, we are not able to postulate what is the most likely ancestral condition that gave rise to bdelloidean asexuality. There are only a few complete mitochondrial DNA sequences available from the Syndermata: *B. plicatilis *(Monogononta [43]), *R. rotatoria *(Bdelloidea; this study) and *L. thecatus *(Acanthocephala [42]). More information, especially from Seisonidea needs to be obtained to confirm the phylogenetic position of Seisonidea within Syndermata. This further phylolgenetic information would be a very substantial ingredient for precisely estimating the origin of asexuality in Bdelloidea and at the same time, contribute to a better understanding of mitochondrial genome evolution within the syndermatan phylogenetic framework.

## Conclusion

In this study, we revisited phylogenetic relationships of Eurotatoria (monophyly versus paraphyly) based on comparative analysis of the complete mitochondrial genome information for major groups of the Syndermata. For this purpose, we determined the first complete mitochondrial genome sequence (15,319 bp) of a bdelloid rotifer, *Rotaria rotatoria*. Comparisons of inferred amino acid sequence and gene arrangement pattern with those of other syndermatan mtDNAs (from the acanthocephalan *L. thecatus *and monogonont *B. plicatilis*) supports the hypothesis that the Bdelloidea shares a most recent common ancestor with Acanthocephala, and not with Monogononta. From this finding, we suggest that the obligatory asexuality of bdelloideans may have secondarily derived from some preexisting condition in earlier lineages of rotifers. Definitively, testing this question of phylogenetic relationships and evolution of different types of life style among the major members of Syndermata awaits further investigation of mitochondrial genome sequencing from Seisonidea.

## Methods

### Sampling and molecular techniques

Rotifers were collected from a small pond at Inha University campus in South Korea (37°26'58.08"N, 126°39'20.09"E) and washed several times with distilled water. Total genomic DNA was extracted from pooled worms using a QIAamp tissue kit (QIAGEN Inc.) and used as a template for PCR amplification. Four small fragments of *Rotaria rotatoria *mtDNA (ranging in size from ~296 to 712 bp) were initially PCR-amplified using corresponding primer sets for each of four gene regions (*cox1 *[LCO1490/HCO2198], *lrRNA *[16SA/16SB], *cob *[Cytb-Uni5-2/Cytb-Uni3-2], and *cox2 *[CO2-Uni5/CO2-Uni3]; see Table 5). PCR reactions were conducted in a total volume of 50 μl reaction mixture containing 0.13 μg/μl of genomic DNA, 10× PCR buffer, 10 mM dNTP mixture, 10 pmole each primer, 25 mM MgCl_2_, and 2.5 units *Taq *polymerase (Roche Co.) using the following cycling conditions: 1 cycle (92°C for 2 min), 35 cycles (92°C for 1 min, 45°C to 55°C for 30 sec, 72°C for 1 min 30 sec), and 1 cycle (72°C for 10 min). The nucleotide sequence determined from these four fragments was then used to design *R. rotatoria *mtDNA-specific primers for long PCR amplification (see also Table 5 for details of the primer information). Four overlapping fragments (ranging in size from 1,559 bp to 5,481 bp) covering the entire mitochondrial genome of *R. rotatoria *were amplified using the Expand Long Template PCR System (Roche, USA) under the following conditions: 1 cycle of 2 min at 94°C (initial denaturation), 35 cycles of denaturation-primer annealing-elongation (10s at 95°C, 1 min at 55°C, and 10 min at 68°C), and 1 cycle of the final extension (10 min at 68°C). A negative control (no template) was included for every PCR run to detect any potential contamination of the PCR products. The amplified PCR products were isolated on a 1.0% agarose gel containing crystal violet, excised and extracted according to the TOPO XL gel-purifying protocol (Invitrogen Co.). Purified DNAs were cloned into *E. coli *competent cells using TOPO XL PCR Cloning kit, as recommended by manufacturer. Sequencing reactions of the target fragments were performed in both directions by 'primer walking' using a Big Dye Terminator Cycle-Sequencing Kit (Applied Biosystems), and overlapping fragments were assembled to complete the sequence of the entire genome.

**Table 5:** Primer sequence information used in this study

Primers	Sequence (5'-3')	Binding region	Source	Estimated size of PCR products
LCO1490	GGTCAACAAATCATAAAGATATTGG	*cox1*	[75]	712 bp
HCO2198	TAAACTTCAGGGTGACCAAAAAATCA	*cox1*		
16SA	CGCCTGTTTATCAAAAACAT	*rrnL*	[76]	437 bp
16SB	CCGGTTGAACTCAGATCA	*rrnL*		
Cytb-Uni5-2	GGATCCGGHTATGTBYTVMYDTGAGG	*cob*	This study	451 bp
Cytb-Uni3-2	GGATCCAYARRAARTAYCATTCWGG	*cob*		
CO2-Uni5	GGWCATCAWTGRTATTGRAVWTATGA	*cox2*	This study	296 bp
CO2-Uni3	TGATTWRCHCCACAVATWTCWGMACA	*cox2*		
RotspCO1+425	GGCTTCATATTGCGGGTGTCTC	*cox1*	This study	1,559 bp
Roti16S-60	TTAGTACGGTCAGATTACTGCAGC	*rrnL*		
Roti16S+320	AGTTGTTTACTACCTCGATGTTGGATC	*rrnL*	This study	3,082 bp
RotaCtB-110	AAAACGTGAAAGAGTAGGAGCACCTACTC	*cob*		
RotaCtB+260	GTGACTTCTATTAATGATAAGGTGGAG	*cob*	This study	4,668 bp
RotaCO2-100	CCGGGATTACAACACGATTATCTACATC	*cox2*		
RotaCO2+180	CGGCAGATGTTCTTCATTCTTGAGCG	*cox2*	This study	5,481 bp
RotspCO1-265	CGAGGAAAAGCCATATCTATCAC	*cox1*		

### Gene Annotation

Twelve protein-coding genes and two ribosomal RNA genes of *R. rotatoria *were identified by sequence comparison with the mtDNA sequences of the acanthocephalan *L. thecatus *and monogonont rotifer *B. plicatilis *and by using the NCBI ORF (open reading frame) Finder. We attempted to find tRNAs using tRNAscan-SE 1.21, but no tRNA-like structure was detected from this search. Therefore, tRNA genes were identified by searching for anticodon consensus motif sequences (TxxxR; xxx = anticodon) and by recognizing potential secondary structures by eye. In this process, we were aided by a web-based automatic annotation program for organellar genomes (DOGMA [64]).

### Phylogenetic analysis

Eleven mitochondrial protein-coding genes (except *atp6 *and *atp8*, which are highly variable in their lengths among the groups) from 35 species (6 ecdysozoans, 22 lophotrochozoans, 5 deuterostomes, and two cnidarian outgroups) representing major clades of the Bilateria, including those of *R. rotatoria *mtDNA, were used in phylogenetic analysis (see Additional file 3 for details of taxon sampling). A multiple alignment of the amino acid sequences for each protein-coding gene was performed using ClustalX [65] with default options. Implementation of ClustalX for multiple sequence alignment does not always guarantee an unambiguous result due to the length and sequence variation among the species. This becomes more problematic when taxon sampling includes a wide range of animal taxa that evolve at very different rates. To improve reliability, we selected conserved blocks from aligned amino acid sequences for each of protein-coding genes using the Gblocks program [66] and a concatenated dataset was then prepared for the subsequent phylogenetic analyses. To reconstruct mitochondrial gene phylogeny, maximum likelihood (ML) and Bayesian inference (BI) were conducted for the concatenated amino acid sequence dataset. For ML analysis, the best-fit model of our amino acid sequence datasets was estimated using the Akaike Information Criterion (AIC) using ProtTest version 2.0 [67]. Maximum likelihood analysis was performed in Treefinder October version [68] using the MtArt matrix [69], which was selected as the best-fit model of amino acid substitution from ProtTest. Nodal support of the resulting ML trees was estimated by nonparametric bootstrap analysis with 500 random replications using Treefinder. We compared the likelihood scores of the competing hypotheses (the best tree versus alternative hypothesis) using various criteria (Expected-Likelihood Weight, ELW [70]; Shimodaira-Hasegawa [71]; Approximately Unbiased, AU [72] tests) implemented in Treefinder. The current version of the MrBayes program does not include the MtArt model of protein sequence, and thus we used the MtRev model with the likelihood parameter setting to "ngammacat = 4", "rates = invgamma" as an alternative best-fit model for Bayesian analysis. The analysis was run for 10^6 ^generations, sampled every 100 generations with four Markov Chain Monte Carlo (MCMC) chains using MrBayes 3.1.2 [73]. Bayesian posterior probability (BPP) values were estimated after the initial 200 saved trees (the first 2 × 10^5 ^generations) were discarded as burn-in. We also conducted maximum parsimony (MP) and neighbour-joining (NJ) analyses and nodal support in the resulting tree was estimated by nonparametric bootstrap analysis with 1,000 random replications in PAUP* 4.0b10 version [74].

## Abbreviations

*atp6*, and *atp8*: genes for ATP synthase subunits 6 and 8; BI: Bayesian inference; bp: base pair; BI: Bayesian inference; BP: bootstrap percentage; BPP: Bayesian posterior probability; *cob*: gene for cytochrome oxidase *b*; *cox1*-*cox3*: genes for cytochrome oxidase *c *subunit 1-3; dNTP: deoxyribonucleotide triphosphate; EST: expressed sequence tag; kb: kilo base; LSU: large subunit nuclear ribosomal DNA; ML: maximum likelihood; MP: maximum parsimony; mtDNA: mitochondrial DNA; *nad1-6*: and *nad4L*: genes for NADH dehydrogenase subunits 1-6 and 4L; NCR: non-coding region; NJ: neighbor joining; nt: nucleotide; ntp: nucleotide pair; ORF: open reading frame; PCR: polymerase chain reaction; *rrnS*: and *rrnL*: genes for small and large mitochondrial ribosomal RNA subunits; SSU: small subunit nuclear ribosomal DNA; tRNA: transfer RNA.

## Authors' contributions

GSM participated in the design of the study and carried out most of the molecular work. JKP designed the study, performed phylogenetic analyses, interpreted the results and drafted the manuscript. Both authors read and approved the final manuscript.

## Supplementary Material

Additional file 1 Predicted secondary structures of the 21 mitochondrial tRNAs of *R. rotatoria*. Click here for file

Additional file 2 **Phylogenetic tree inferred from Bayesian analysis for metazoan mitochondrial amino acid sequences before the exclusion of nematode species from the analysis.** Numbers above/below branches are Bayesian posterior probability (BPP) and local rearrangement-expected likelihood weight (LR-ELW) edge support estimated from Bayesian and maximum likelihood analyses, respectively (BPP/LR-ELW). The branches that are supported with values of ≤50% or not consistent between Bayesian and maximum likelihood methods in their positions are represented by "-". Click here for file

Additional file 3 **Taxa/species used for phylogenetic analysis in this study.** The Chaetognatha was traditionally considered a member of deuterostomes, but recent molecular analysis using SSU rDNA sequence suggested that they are not deuterostomes [77,78]. Although phylogenetic position of Chaetognatha with other metazoan phyla still remained enigmatic, recent molecular phylogenetic surveys including comparative analysis of the complete mitochondrial genome often suggest its affinity to protostomes (for more details, see [21,79-81]). Therefore, we tentatively included two chaetognath species (*Spadella cephaloptera *and *Paraspadella gotoi*) in the Protostome clade for phylogenetic analysis. Click here for file
