# Supplementary material for: Eurotatorian paraphyly: Revisiting phylogenetic relationships based on the complete mitochondrial genome sequence of Rotaria rotatoria (Bdelloidea: Rotifera: Syndermata)
Source: BMC Genomics. 2009 Nov 17;10:533. doi: 10.1186/1471-2164-10-533 (PMC2784805; doi:10.1186/1471-2164-10-533)
Supplement: Additional file 3 — Taxa/species used for phylogenetic analysis in this study. The Chaetognatha was traditionally considered a member of deuterostomes, but recent molecular analysis using SSU rDNA sequence suggested that they are not deuterostomes [77,78]. Although phylogenetic position of Chaetognatha with other metazoan phyla still remained enigmatic, recent molecular phylogenetic surveys including comparative analysis of the complete mitochondrial genome often suggest its affinity to protostomes (for more details, see [21,79-81]). Therefore, we tentatively included two chaetognath species (Spadella cephaloptera and Paraspadella gotoi) in the Protostome clade for phylogenetic analysis. [file 1471-2164-10-533-S3.DOC]

**Additional File 3**

Taxa/species used for phylogenetic analysis in this study

| Taxon/Species | Classification | Accession  number |
| --- | --- | --- |
| Radiata |  |  |
| *Acropora tenuis* | Cnidaria, Anthozoa | NC_003522 |
| *Aurelia aurita* | Cnidaria, Scyphozoa | NC_008446 |
| Lophotrochozoa |  |  |
| *Nautilus macromphalus* | Mollusca, Cephalopoda | NC_007980 |
| *Katharina tunicata* | Mollusca, Polyplacophora | NC_001636 |
| *Clymenella torquata* | Annelida, Polychaeta | NC_006321 |
| *Lumbricus terrestris* | Annelida, Clitellata | NC_001673 |
| *Urechis caupo* | Echiura | NC_006379 |
| *Sipunculus nudus* | Sipunculida | NC_011826 |
| *Terebratalia transversa* | Brachiopoda | NC_003086 |
| *Terebratulina retusa* | Brachiopoda | NC_000941 |
| *Loxosomella aloxiata* | Entoprocta | NC_010432 |
| *Loxocorone allax* | Entoprocta | NC_010431 |
| *Phoronis psammophila* | Phoronida | AY368231 |
| *Bugula neritina* | Bryozoa | NC_010197 |
| *Flustrellidra hispida* | Bryozoa | NC_008192 |
| *Spadella cephaloptera* | Chaetognatha | NC_006386 |
| *Paraspadella gotoi* | Chaetognatha | NC_006083 |
| Platyzoa |  |  |
| *Hymenolepis diminuta* | Platyhelminthes, Cestoda | NC_002767 |
| *Fasciola hepatica* | Platyhelminthes, Trematoda | NC_002546 |
| *Gyrodactylus salaris* | Platyhelminthes, Monogenea, Monopisthocotylea | NC_008815 |
| *Microcotyle sebastis* | Platyhelminthes, Monogenea, Polyopisthocotylea | NC_009055 |
| *Leptorhynchoides thecatus* | Acanthocephala | NC_006892 |
| *Rotaria rotatoria* | Rotifera, Bdelloidea | GQ304898 |
| *Brachionus plicatilis* | Rotifera, Monogononta | NC_010484, NC_010472 |
| Ecysozoa |  |  |
| *Limulus polyphemus* | Arthropoda, Chelicerata | NC_003057 |
| *Lithobius forficatus* | Arthropoda, Myriapoda | NC_002629 |
| *Panulirus japonicus* | Arthropoda, Crustacea | NC_004251 |
| *Drosophila melanogaster* | Arthropoda, Hexapoda | NC_001709 |
| *Epiperipatus biolleyi* | Onychophora | NC_009082 |
| *Priapulus caudatus* | Priapulida | NC_008557 |
| *Trichinella spiralis* | Nematoda, Enoplea | NC_002681 |
| *Caenorhabditis elegans* | Nematoda, Chromadorea | NC_001328 |
| Deuterostomia |  |  |
| *Branchiostoma lanceolatum* | Cephalochordata | NC_001912 |
| *Strongylocentrotus purpuratus* | Echinodermata | NC_001453 |
| *Balanoglossus carnosus* | Hemichordata | NC_001887 |
| *Xenopus laevis* | Chordata, Vertebrata, Amphibia | NC_001573 |
| *Acipenser dabryanus* | Chordata, Vertebrata, Actinopterygii | NC_005451 |
